# Community Coalitions for Change and the Policy, Systems, and Environment Model: A Community-Based Participatory Approach to Addressing Obesity in Rural Tennessee

**DOI:** 10.5888/pcd16.180678

**Published:** 2019-09-05

**Authors:** Heather Sedges Wallace, Karen L. Franck, Cori L. Sweet

**Affiliations:** 1 University of Tennessee Institute of Agriculture, Family and Consumer Sciences, Knoxville, Tennessee

## Abstract

Four rural counties in Tennessee adopted the policy, systems, and environment (PSE) approach to address the obesity epidemic in their communities. The community-based participatory initiative, Community Coalitions for Change (C3), was embraced by 67,400 community members and 67 organizations. During year 1, coalition members discussed a need to return to long-held traditions of collective community engagement and action to address rural obesity rates. In response, C3 established 25 community gardens and supported 10 existing gardens, resulting in 8,300 community members who received garden produce. Sites began with an average number of 11 physical activity resources, which increased by year 3 to an average of 13 resources as a result of C3 activities. Overall, 61% (248 of 405) of survey respondents participating in direct education programs reported being more physically active as a result of participating in the programs, 59% (117 of 199) reported eating more fruit, and 66% (131 of 199) reported eating more vegetables. Implications for public health include timing and aligning obesity prevention activities with ongoing initiatives that are working toward similar goals.

**Summary**

**What is already known on this topic?**

Complex health issues such as obesity are best addressed through interventions that operate at various levels of behavior change (eg, individual, community, cultural). These interventions are most successful when implemented at the community level with diverse groups working together to achieve change.

**What is added by this report?**

Four rural counties in Tennessee adopted the policy, systems, and environment (PSE) approach to address the obesity epidemic in their communities. Community-based participatory practice was the guiding force in conducting activities. The community-based participatory initiative was embraced by 67,400 community members and 67 organizations.

**What are the implications for public health practice?**

These interventions have been effective in rural communities where health care resources are often limited. Key to this transformative approach is timing and alignment with ongoing initiatives working toward similar goals.

## Introduction

Complex health issues such as obesity are best addressed through interventions that address various levels of behavior change (eg, individual, community, cultural). These interventions are most successful when implemented at the community level with diverse groups working together to achieve change. Four rural counties in western Tennessee (Haywood, Humphreys, Lake, and Lauderdale) with adult obesity rates greater than 40% (on the basis of 2012 Behavioral Risk Factor Surveillance System data) participated in a community-based intervention to reduce obesity rates. The initiative, Community Coalitions for Change, or C3, began with the goal of reaching the 67,400 community members who were at disproportionate risk for chronic diseases associated with obesity, poor nutritional habits, and lack of physical activity. Community advisory councils, established before the C3 initiative, had identified obesity as a top priority at least a decade earlier. Thus, public health surveillance and community-based perspectives aligned on the need and rationale for the C3 intervention. State-level faculty and specialists affiliated with the Family and Consumer Sciences (FCS) department of the University of Tennessee Institute of Agriculture Extension (Extension) conceptualized the approach, guided implementation, and conducted evaluations of the C3 initiative. FCS agents and C3 program assistants collaborated with 19 groups, including local health department councils, the Tennessee Department of Education’s Coordinated School Health councils, networks dedicated to preventing substance abuse, state and county commissions on aging, and several county and state park boards. During the 4 years of the intervention, 160 people representing 67 organizations served on C3 coalitions (Appendix).

## Purpose and Objectives

The purpose of this initiative was to engage communities in the process of reducing the prevalence of obesity over the long term and in accordance with the policy, systems, environment (PSE) model. The public health community embraces the PSE approach (1–3). This model stresses the importance of direct education and recognizes the need to alter contexts that influence personal health behaviors.

PSE changes described herein build on the foundation laid by previously funded Community Transformation Grant programs led by community coalitions in partnership with local health departments and other community-based organizations (4). Our work expanded the scope and scale of those programs by focusing on instigating community-wide cultural, social, and behavioral changes rather than individual-level behavior.

All 4 counties participating in C3 activities are rural and have a long agricultural history and a county-based Cooperative Extension infrastructure. Across all 4 counties, the median annual income is $34,563 (5), an average 27% of households live below the federal poverty level, and an average 20% of the population reports being food insecure (6). Three of the 4 counties are predominately non-Hispanic white, and all have strong faith-based communities.

## Intervention Approach

Community-based participatory practice (CBPP) was the guiding force in conducting C3 activities. This approach uses community engagement and empowerment to improve outcomes (7,8). It involves building relationships between programs and community members and focuses on developing mutual trust and equality; program participants and community members are viewed as important contributors to the entire process (9). These relationships are developed and maintained throughout the process, from identifying critical issues of concern cited by the community to disseminating results.

Evidence of the CBPP model, and a key driver to implementation success, was that C3 coalitions were born out of, or modeled on, existing health councils in all 4 counties. Those groups consisted of representatives from local community groups, businesses, organizations, and FCS agents. C3 coalitions provided direction on grant activities. These activities included identifying and engaging new coalition members, working on needs assessment activities, prioritizing grant activities, working together on intervention projects, and identifying opportunities for sustainability and potential to expand grant activities. Community members also provided ongoing feedback to the program about what was working and what was not working. This feedback permitted an intervention that was responsive to community needs.

With the support of FCS agents and C3 program assistants, communities implemented projects in years 2 through 4. Prioritized intervention activities were in the following areas: 1) increasing the number of direct educational programs delivered through Extension, 2) increasing interventions that promoted healthy nutrition options, and 3) increasing physical activity interventions that promoted exercise and being active. More than $3 million was dedicated to these projects, and each county had equal access to funds at the start of the program. Haywood County used the most funding, followed by Lauderdale, Humphreys, and Lake counties, in that order. Because the process of allocating and spending funds was transparent — counties were equally allocated at outset, and decisions on how and why to spend the funds were made by each community — we had no problems in allocating funds.

## Evaluation Methods

During the first year, FCS evaluation staff members (ie, the evaluation team) completed a comprehensive situational analysis for each county to identify community needs and strengths. After approval from the University of Tennessee’s institutional review board, the evaluation team collected input from community members through surveys and focus groups and worked with county FCS agents and C3 program assistants to complete assessments of parks and retail food venues. The evaluation team used the Physical Activity Resource Assessment (10) to complete recreational site audits in the 4 counties. The evaluation team then examined existing data, including recent community needs assessments (conducted within the last 5 years), census data, health department reports, and data available through geographic resource mapping at CommunityCommons.org.

In years 2 through 4, the lead evaluation specialist reviewed data from surveys, interviews, focus groups, audits, and pedometer monitoring in both process and outcome evaluation activities. The evaluation team determined appropriate evaluation methods on the basis of how to access and collect information in a community-based manner without exhausting communities with repetitive efforts. In year 4, the final year of the grant, the evaluation team also engaged 40 coalition and community members in the process of ripple effect mapping (REM). REM is a facilitated group process based on the Community Capitals Framework to collect qualitative data about perceived outcomes and sustainability efforts related to projects like C3 (11).

## Results

**Direct education.** Overall, 1,844 adults, children, and adolescents participated in direct education opportunities such as in-store food demonstrations, cooking classes, gardening workshops, nutrition programs, and exercise classes. Of these, 405 (22%) completed surveys about physical activity and 199 (11%) completed surveys about healthy eating. For physical activity, 61% (248 of 405) reported being more physically active as a result of participating in the programs. For healthy eating, 59% (117 of 199) reported eating more fruit and 66% (131 out of 199) reported eating more vegetables.

**Nutrition interventions.** All 4 counties had a strong focus on promoting healthy food choices. Related work aligned to the PSE model in various ways. Among these are 1) policy changes allowing children to carry water bottles at school; 2) systemic shifts toward collaboration between organizations, evidenced by providing community garden vegetables at the local food pantry; and 3) environmental alterations such as promotional and motivational signage in restaurants, grocery stores, and corner stores along with the installation of food storage and display equipment.

Intercept surveys designed to gauge familiarity with the intervention among C3-participating grocery store customers showed a range of responses among 162 respondents, from 38% (n = 61) who recalled seeing the bundled promotions to 54% (n = 87) who recalled seeing the “shelf-talkers” (branded, nutritional information attached to a store shelf to capture consumers’ attention and increase awareness and knowledge about an item). Almost one-third (n = 43) of respondents indicated that these promotions encouraged them to choose healthier foods. Additionally, interviews with 8 retail food managers revealed that 7 managers felt that the interventions had been successful and 4 managers felt that the interventions had improved their sales.

**Physical activity interventions.** The third priority was physical activity interventions that promoted exercise and being active. We found evidence of the PSE model in policy changes. For example, 9 churches opened their indoor and outdoor facilities (eg, gymnasiums, sports fields) to noncongregation members, and 11 schools permitted use of their walking paths or playground equipment. However, the fear of liability and a type of cultural aversion to signing official documents precluded institutions from committing these neighborly practices to paper. C3 increased communities’ capacity for systems change by promoting walking clubs at senior living facilities. The environmental context was the area of greatest change related to physical activity. Promotional signage was created by state-level content experts in partnership with a contracted marketing firm and then installed in 53 venues in all 4 counties. Four new community parks were created, and physical activity equipment was installed in 38 venues.

In year 1, the evaluation team assessed 36 park and recreation sites by using the Physical Activity Resource Assessment. In years 2 through 4, only the 26 sites that were selected by coalitions for improvements were assessed by using the Physical Activity Resource Assessment. In year 1, sites had an average number of 11 physical activity resources, which increased by year 3 to an average of 13 resources as a result of C3 activities. Most of these changes related to bike racks, adult exercise stations, and sports equipment and courts.

**Integrated PSE outcomes and collective impact.** The inherent nature of PSE work is synergistic, meaning that one alteration intends to promote change in another arena, such as the way in which a policy change affects how systems function and/or permits change to the environmental context. For example, availability of healthy food preparation equipment catalyzed the adoption of nutrition-related policy changes in 6 churches, where they replaced some foods with healthier options (eg, fried chicken replaced by grilled chicken). Two school systems agreed to implement a policy that allowed students to bring water bottles into the academic setting after C3 provided water bottle refilling stations. In Lake County, the Coordinated School Health representative implemented a change in school policy that led to banning unhealthy food as rewards to students.

During the situational analysis in year 1, coalition members discussed a need to return to long-held traditions of collective community engagement and action related to increasing access to healthy foods. In response, C3 established 25 community gardens and supported 10 existing gardens. More than 8,300 community members, including students, seniors, subsidized housing residents, and food pantry clients, received produce from these gardens. Gardens were also successful in engaging volunteers: 632 volunteers donated 6,188 hours in years 3 and 4 for a value of $152,341. In addition, 37 laborers donated 350 hours, and $3,790 of donated supplies were received.

Farmers markets were another method through which community members built on their shared value of collective impact. Two counties (Humphreys and Lauderdale) worked with their existing farmers markets to encourage community members to purchase locally grown fruits and vegetables. REM participants identified the mutual benefit the market has for farmers and participants. They reported that the market was well attended and sold out of produce occasionally. REM participants credited C3-affiliated efforts for increased farmers market participation and revenue.

## Implications for Public Health

CBPP has been used extensively to address complex health issues such as obesity prevention (12), physical activity in rural communities (13), and chronic diseases (14). CBPP facilitates action and change at the individual, family, and community levels, which is necessary for obesity prevention. In addition, CBPP allows researchers to explore issues that affect health outcomes and to define novel and creative ways to reduce health disparities. CBPP has been effective in rural communities where health care resources are often limited (15,16), and it was effective in our initiative. Critical to CBPP are meaningful engagement, ownership of interventions, accountability, ability to build on strengths, and willingness to recognize and respect that a well-intentioned intervention is not succeeding. The CBPP approach embraced by the C3 initiative empowered community members to sustain interventions as they improved their own health outcomes and began to transform their communities.

Key to this transformative approach is timing and alignment with ongoing initiatives working toward similar goals. The confluence of the C3 grant with the Governor’s Foundation for Health and Wellness initiative, Healthier Tennessee, and the Tennessee Department of Health’s Primary Prevention Initiative, was mutually beneficial. Many of the activities that helped counties achieve Healthier Tennessee status also helped accomplish C3 goals — and vice versa. In exactly the same way, health department employees were able to participate in C3 projects, while meeting their own agency’s Primary Prevention Initiative goals. This synergistic outcome was reiterated by REM participants who spoke about the many important obesity prevention outcomes that were facilitated by these overlapping and interlocking efforts.

## Appendix Organizations Involved in Community-Based Participatory Approach to Addressing Obesity in Rural Tennessee

• African Methodist Episcopal minister

• Afterschool care

• Area health education center

• Arts council

• Baptist minister

• Board of education

• Boys and girls club

• Chamber of commerce

• Child care provider

• Children’s hospital

• Church of Christ minister

• City government administration

• City mayors

• City parks and recreation

• City police department

• Community centers

• Community hospital

• Community park association

• Coordinated school health

• Corner store manager

• County government administration

• County health department

• County mayors

• County parks and recreation

• County school system

• Department of children’s services

• Department of corrections

• Department of human services

• Department of transportation

• Economic development council

• Extension 4-H agents

• Extension agriculture agents

• Extension family and community education volunteer clubs

• Family life center

• Farmers

• Farmers’ market administrators

• Federally qualified health center

• Governor’s Foundation for Health and Wellness

• Grocery store manager

• Head Start

• Hospital community outreach program

• Manufacturing business

• Master gardeners

• Medicaid coordinator

• Mental health services

• Methodist minister

• National alliance on mental illness

• Neighborhood association

• Outpatient drug treatment center

• Physical therapy center

• Pregnancy center

• Private counseling center

• Private gym

• Private insurance company

• Private weight-loss clinic

• Regional commission on children and youth

• Senior center

• Sheriff’s department

• State commission on children and youth

• State health department

• State health insurance assistance program

• State parks

• Technical college

• Teen job development program

• Tennessee General Assembly

• University

• YMCA
